# Supplementary material for: Quantifying the impact of scholarly papers based on higher-order weighted citations
Source: PLoS One. 2018 Mar 29;13(3):e0193192. doi: 10.1371/journal.pone.0193192 (PMC5875758; doi:10.1371/journal.pone.0193192)
Supplement: S1 Data Source — (DOCX) [file pone.0193192.s001.docx]

S1 Data Source

This study in paper applies APS data set and Google Map, and they may be accessed from:

APS Data Set:

<https://journals.aps.org/datasets>

(click the button” Request Access”)

Google Map API

[https://maps.googleapis.com/maps/api/geocode/json?address=AffiliationName&key=%3c<YourKey>](https://maps.googleapis.com/maps/api/geocode/json?address=AffiliationName&key=%3c%3cYourKey%3e)

(note: an individual key may be requested from Google, which replaces <YourKey> in the link above)
